# Protocol to generate a microfluidic vessels-on-chip platform using human pluripotent stem cell-derived endothelial cells

**DOI:** 10.1016/j.xpro.2024.103300

**Published:** 2024-09-07

**Authors:** Caroline Remmert, Munkhtur Otgonbayar, Julius Alexander Perschel, Maren Marder, Matthias Meier

**Affiliations:** 1 Helmholtz Pioneer Campus, Helmholtz Zentrum München, 85764 Munich, Germany; 2 Center for Biotechnology and Biomedicine, University of Leipzig, 04103 Leipzig, Germany

**Keywords:** Cell culture, Stem Cells, Tissue Engineering, Biotechnology and bioengineering

## Abstract

Here, we present a protocol for producing a microfluidic vessel-on-chip platform using human pluripotent stem cell-derived endothelial cells (SC-ECs). We describe steps for manufacturing the 3D-printed chip, cell culturing to generate SC-ECs, hydrogel patterning, and the formation and cultivation of barrier-forming vessels. We then detail procedures for the retrieval of cells and media from the open microfluidic chip platform to enable multi-omics analysis.

For complete details on the use and execution of this protocol, please refer to Marder et al.^1^

## Highlights

• Establishment of a microfluidic chip platform for stem cell-derived endothelial vessels
• Protocol for chip manufacturing and generation of vessels-on-chip
• Extraction of cells from the chip allows multi-omics analysis of vessels-on-chip

## Abstract (editor highlights)

Publisher’s note: Undertaking any experimental protocol requires adherence to local institutional guidelines for laboratory safety and ethics.

## Graphical abstract

**Figure:**
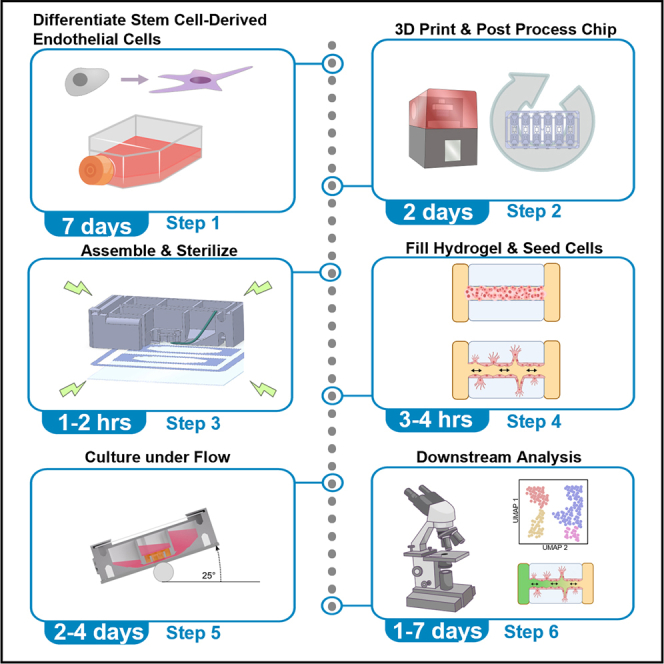

## Before you begin

**Figure 1:**
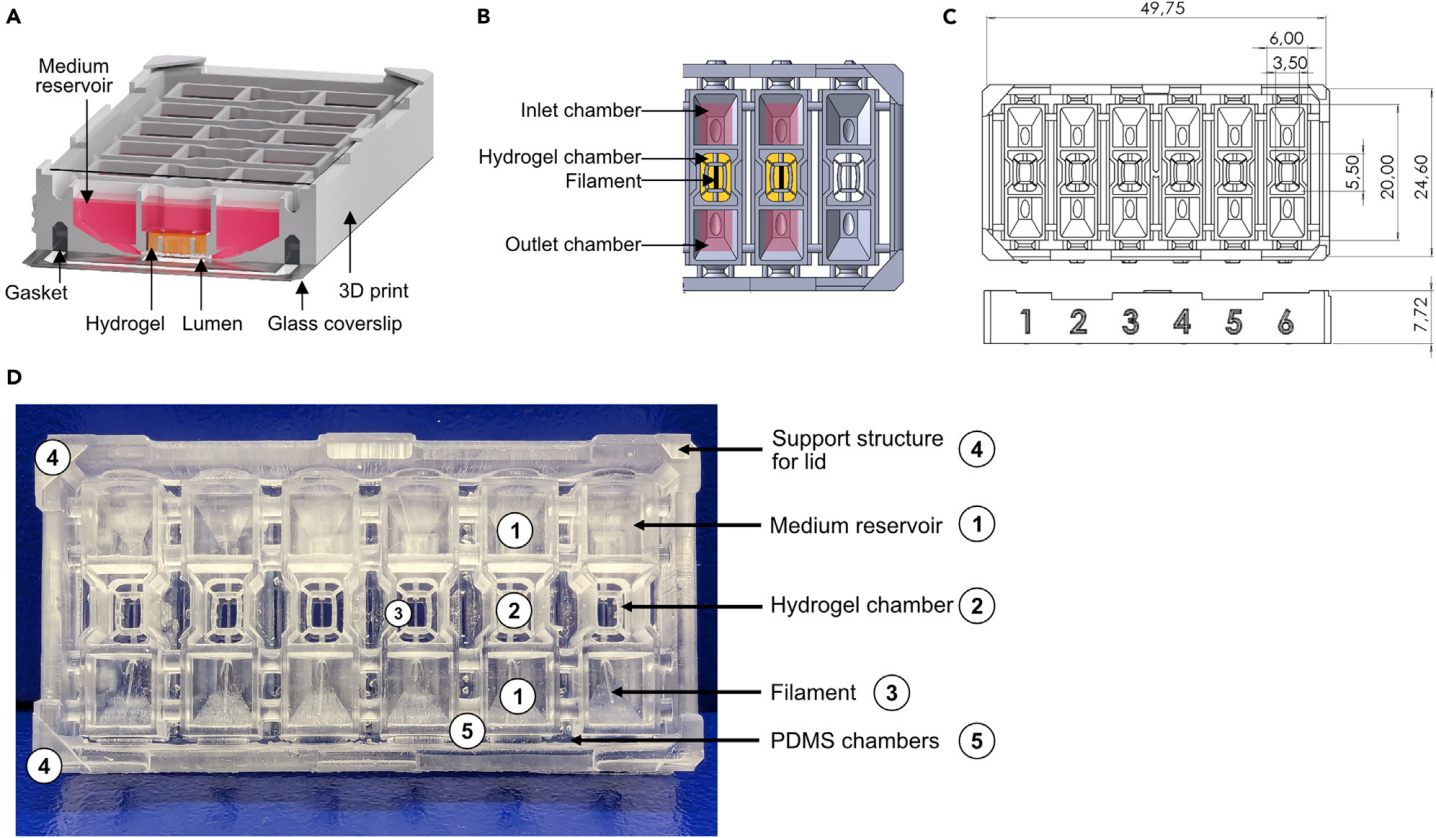
The open microfluidic chip platform for organotypic cell cultures (A) Cross-section view and (B) top view of the fully assembled vessel-on-chip. The culture media and patterned hydrogel are denoted in pink and yellow, respectively. (C) Dimensions of the 3D printed chip platform in cm. (D) Assembled chip with cross reference numbers for the protocol. Figure reprinted and adapted with permission from Marder and Remmert et al., 2024.^1^

This protocol outlines the comprehensive steps for engineering stem cell-derived vessels-on-chip. We designed an organ-on-chip platform in which human induced pluripotent stem cell-derived endothelial cells (SC-ECs) form a vessel within a hydrogel-patterned microchannel. The platform is manufactured using a stereolithography 3D printer, allowing for rapid prototyping and design adaptation. During assembly, the 3D-printed plastic body is bonded to an optical glass substrate with adhesive foil. The chip comprises six units, each containing a hydrogel chamber and two medium reservoirs. Using a nylon filament, hydrogels with variable compositions can be patterned with straight microfluidic channels ranging from 50 to 350 μm in diameter (see Figure 1). Within this setup, vessels are generated in a collagen type I hydrogel, but the platform is not limited to this hydrogel composition. After seeding, cells are cultured under gravity-induced flow conditions, leading to vessel formation within 24–48 h. The open design of the chip enables long-term culturing, cell stimulation, and the simple extraction of cell material for downstream analysis. Due to the short working distance of the tissue to the substrate, high-resolution imaging of whole mount stainings can be performed with a confocal microscope. In addition to standard immunofluorescence analysis on whole mounts, the chip enables the retrieval of the whole tissue for cryosectioning. Compared to other organ-on-chip platforms, the high yield of cell and media retrieval from the chip allows for single-cell RNA sequencing (scRNA-seq) or proteomic mass spectrometry analysis. The stem cell-derived vessel-on-chip offers the opportunity to study *in vitro* endothelial development, tissue-specific toning, and patient-derived disease models at high resolution and single-cell levels (see Marder and Remmert et al.^1^). Combined with the open hydrogel design, our setup facilitates the seamless engineering of vessels.

### Printer requirements

To produce the vessels-on-chip plastic body, a digital light projection (DLP) printer with a pixel resolution in yx and z direction of at least 27 μm and 50 μm, respectively, is required.

### Differentiation of hiPSCs to SC-ECs

**Timing: 7 days**

**Figure 2:**
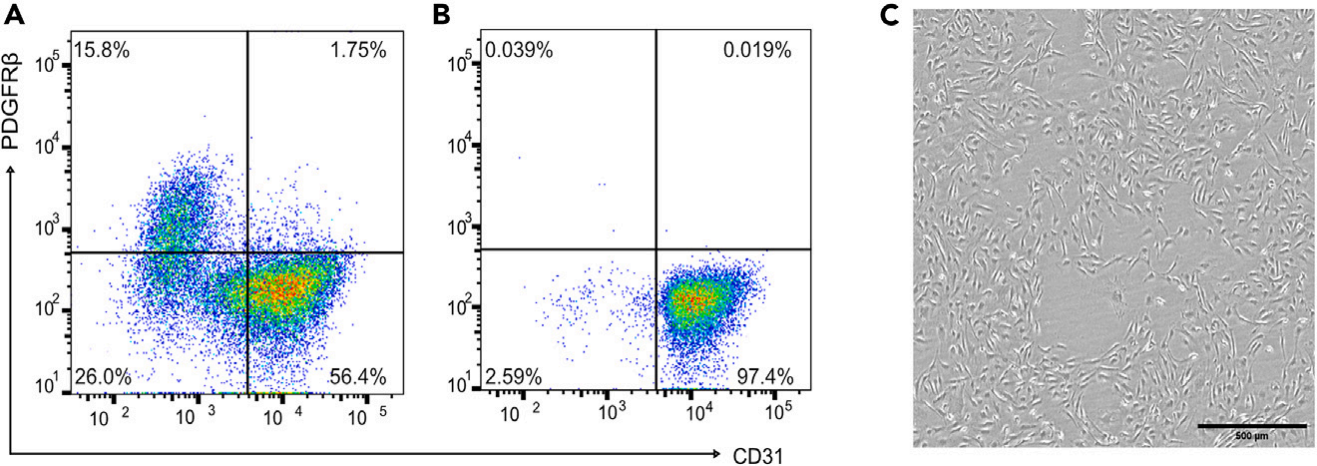
Differentiation of human induced pluripotent stem cells (hiPSC) into stem cell-derived endothelial cells (SC-ECs) (A) Representative flow cytometry analysis from a differentiation experiment following the protocol by Marder and Remmert et al.^1^ On average, the differentiation protocol yields 50 ± 10% and 20 ± 10% expressing CD31 and PDGFRβ cells, respectively. (B) Flow cytometry analysis of a representative SC-EC culture after sorting for the endothelial marker CD31. (C) Example bright field image of SC-ECs cultured on fibronectin-coated plates. Scale bar, 500 μm.

Before you begin, stem cell-derived endothelial cells (SC-ECs) need to be differentiated from human induced pluripotent stem cells (hiPSCs). This protocol uses a differentiation protocol that is published in Marder and Remmert et al.^1^ and describes an adopted protocol from previous publications.^2^^,^^3^^,^^4^ In brief, hiPSCs are differentiated to SC-ECs in 6 days. For the differentiation, hiPSCs are transferred into a 3D cell culture format and cultured on an orbital shaker in a cell incubator with a rotation frequency of 100 rpm. From day zero to day three of differentiation N2B27 medium supplemented with BMP4 (25 ng/mL), and CHIR 99021 (7.5 μM) is used. From day three to six of differentiation, StemPro-34 complete medium supplemented with VEGFA (200 ng/mL), and forskolin (2 μM) is used. After successful differentiation on day 6, SC-ECs can be sorted by flow cytometry for a SC-EC population >90% (Figure 2, antibodies for sorting are provided in the key resources table).

**Pause point:** Differentiated SC-ECs can be cryo-preserved for later experiments following standard protocols. To do this, 1 million cells per ml can be cryopreserved in FBS with 10% DMSO.

**Figure 3:**
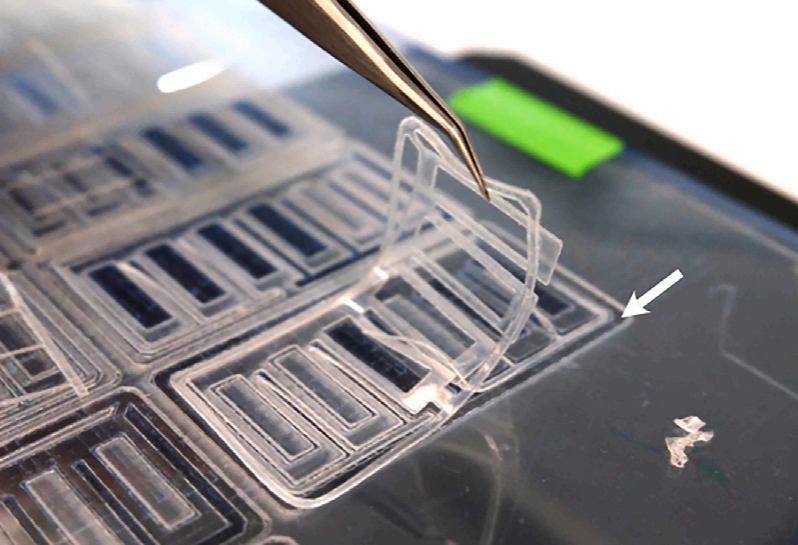
Preparation of the adhesive foils by laser cutting White arrow points to protective foil that is not cut entirely through to simplify the handling.

**Figure 4:**
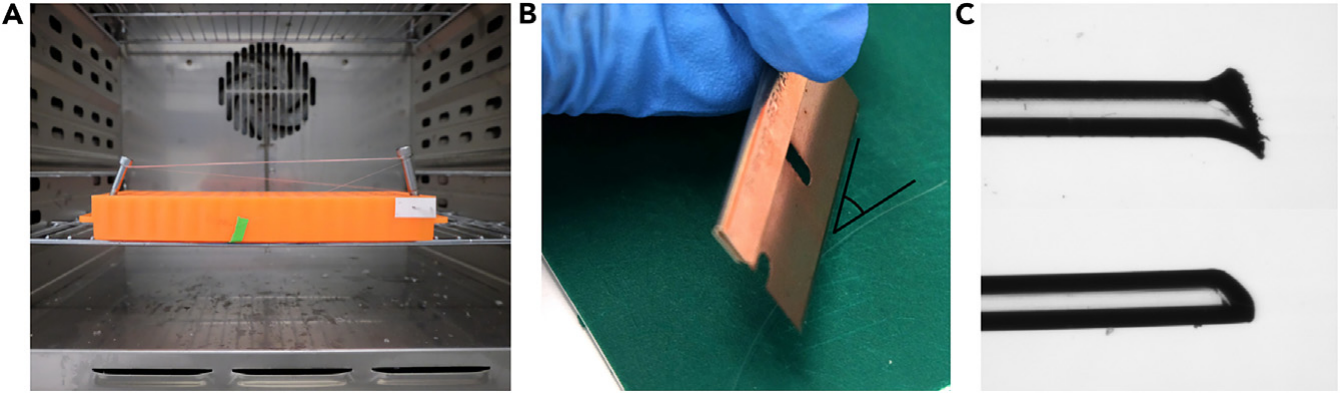
Preparation of the filaments (A) Straighten the filament by stretching it on a straight object and placing it at 80°C for 10 min. (B) Place the filament on a cutting board and cut it at a 45° angle to a length of approximately 16 mm. (C) A blunt blade will produce an unclean cut (top) which can rupture the hydrogel. A sharp blade is required to obtain a clean cut (bottom).

### Preparation of adhesive foils

***Note:*** The adhesive foil was cut with a Glowforge Plus laser cutter, as its resolution is high enough to cut intricate shapes while being manageable and easy to handle.

1. Download the template for cutting the adhesive foil from our GitHub (“AdhesiveFoil_CutFile.svg”).
  a. GitHub: https://github.com/MeierLabMiBioEng/Protocol-to-generate-stem-cell-derived-vessels-on-chip.
  b. Zenodo: https://zenodo.org/doi/10.5281/zenodo.12742938.
2. Adhere the double-sided adhesive foil to a substrate, such as a flat wooden or plastic board, using tape on the edges.

***Note:*** Ensure the substrate is flat, as curved boards can cause irregularities in cut depth.

3. Set the laser cutter speed to 250 and the power to 20.

***Note:*** Setting the power to 23 will cut through all three layers of the foil. Cutting only two layers (the sticky foil and protective film) makes handling and assembly much easier (Figure 3).

### Preparation of nylon filaments

**CRITICAL:** Nylon filaments should be cut to length using a new, sharp blade on a flat cutting board. Using old or dull blades can result in torn filaments, which damage the hydrogel upon its removal. Additionally, straightening the filaments beforehand will result in straighter channels.

4. Stretch the filament by wrapping it around a straight object, such as a microtube rack, and placing it at 80°C for 10 min (Figure 4A).
5. Allow the filament to cool down to room temperature (RT, 20°C–25°C).
6. Place the filament on a cutting board and cut it at a 45° angle to a length of approximately 16 mm (Figures 4B and 4C).
7. Inspect the cut under a microscope to ensure it is clean (Figure 4C).

## Key resources table

**Table:** 

REAGENT or RESOURCE	SOURCE	IDENTIFIER
**Antibodies**		

CD31-FITC (20 μL per 1 × 10^6^ cells)	BD	Cat#555445
CD140b-PE (20 μL per 1 × 10^6^ cells)	BD	Cat#558821

**Chemicals, peptides, and recombinant proteins**		

10× PBS	Invitrogen	AM9624
Accutase	Sigma-Aldrich	A6964
Agarose	Carl Roth	2267.4
Collagen type I	Corning	CLS354236
Collagenase P	Roche	11213857001
EGM-2 bullet kit with supplements	Sigma-Aldrich	C-22011
Ethanol	Sigma-Aldrich	1009831000
Fetal bovine serum (FBS)	Sigma-Aldrich	F7524
Fibronectin from bovine plasma	Thermo Fisher Scientific	33010018
Fluorescein isothiocyanate-dextran	Sigma-Aldrich	FD40-100MG
GlutaMAX supplement	Life Technologies	10564011
Human FGF2	Miltenyi Biotec	130-093-838
Isopropanol	Sigma-Aldrich	33539-1L-M
L-glutamine (200 mM)	Thermo Fisher Scientific	25030024
NaOH	Carl Roth	P031.2
PBS−/−	Gibco, Thermo Fisher Scientific	14190094
Penicillin-streptomycin	Gibco, Thermo Fisher Scientific	15140122
Polydimethylsiloxane (PDMS) SYLGARD 184 silicone elastomer kit	Dow	1317318
Pro3dure GR-10 resin (transparent)	Litholabs	HX10031
Recombinant human VEGFA_165_	PeproTech	100-20-250
ROTI-Histofix (4% PFA)	Carl Roth	P087.6
StemPro-34	Thermo Fisher Scientific	10639011
UltraPure distilled water (ddH_2_O)	Invitrogen	10977035

**Deposited data**		

Supplemental STL files	This paper; Zenodo: https://doi.org/10.5281/zenodo.12742938	GitHub: https://github.com/MeierLabMiBioEng/Protocol-to-generate-stem-cell-derived-vessels-on-chip

**Experimental models: Cell lines**		

Human induced pluripotent stem cells (HiPSC)	HMGUi001-A	https://hpscreg.eu/cell-line/HMGUi001-A

**Software and algorithms**		

Asiga Composer	Asiga	https://www.asiga.com/software-composer/
SOLIDWORKS 2022	Dassault Systèmes	https://www.solidworks.com

**Other**		

Asiga Pico 2 HD	Asiga	UV385
Confocal microscope Zeiss LSM 880	Zeiss	LSM880
Coverslip 24 × 50 mm #1,5	VWR	630-2602
Double-sided adhesive foil	ARcare	90445Q
Filter screen 190 μm	Würth	0899700110
Glowforge Plus	Glowforge	N/A
Multipette	Eppendorf	4987000029
Nylon filament 150 μm	efco creative GmbH	1005215
OrganoFlow L (rocking shaker)	Mimetas	MI-OFPR-L
Otoflash G171	NK-Optik	644900
Petri dish, 145/20 mm, with vents	Greiner Bio-One	639102
pH-indicator strip 5.5–9.0	Merck	90105354
Stage top incubator	Tokai Hit	STX
Stirrup-shaped blades type 210	Carl Roth	CK07.2
Scalpel disposable sterile, 11	Carl Roth	1PYT.1
Table-top UV sterilizer	Custom	N/A
TC20 automated cell counter	Bio-Rad	CA# 1450102
Tweezer 3CBS	Weller Erem	3650896
Ultrasonic cleaner USC - TH	VWR	142-0087

## Materials and equipment

In the following sections, we provide the recipes for growth factors, cell culture media, and other solutions required for the protocol. Additionally, we outline the initial material preparation steps for chip manufacturing.

### Preparation of VEGFA

**Timing: 30 min**

• Lyophilized VEGFA (recombinant human VEGFA_165_) is purchased in a packaging size of 100 μg.
• Centrifuge the VEGFA tube at 13,000 rpm for 30–60 s at RT.
• Reconstitute VEGFA in 1 mL sterile ultrapure water (ddH_2_O) to obtain a 100 μg/mL stock concentration.
• Prepare 20 μL aliquots in 1.5 mL Eppendorf tubes, label, and store at −20°C for up to 12 months.
• Once thawed, aliquots can be stored at 4°C for 1 week.

### Preparation of FGF2

**Timing: 30 min**

• Lyophilized human FGF2 is purchased in a packaging size of 50 μg.
• Reconstitute FGF2 in 500 μL sterile ddH_2_O to obtain a 100 μg/mL stock concentration.
• Prepare 20 μL aliquots in 1.5 mL Eppendorf tubes, label, and store at −20°C for at least 6 months.

***Note:*** The exact expiration date for FGF2 is provided by the vendor.

• Once thawed, aliquots can be stored at 4°C for 1 week.

### Preparation of fibronectin coating solution

• Stock solutions: Fibronectin from bovine plasma is dissolved to a concentration of 1 mg/mL in ddH_2_O.
  ○ To prepare 1 mL of stock solution, reconstitute 1 mg of lyophilized fibronectin in sterile ddH_2_O.
  ○ Prepare 60 μL aliquots in 1.5 mL Eppendorf tubes, label them, and store at −20°C for up to 12 months.
• Coating solution: For SC-EC cultures, fibronectin stock solutions are diluted with sterile PBS −/− to a final concentration of 5 μg/mL.
  ○ To prepare 12 mL of the coating solution, dilute 60 μL of stock solution (1 mg/mL) in 12 mL PBS −/−.
  ○ **Note:** PBS−/− can be used at RT.
  ○ The coating solution can be stored at 4°C for 1 month.

### StemPro34 supplemented medium

For the SC-EC cell culture, StemPro34 complete medium supplemented with 100 ng/mL VEGFA, 100 ng/mL FGF2, and 15% FBS is used. The preparation of this medium is described below.

First, prepare the StemPro34 complete medium.

**Table:** StemPro34 complete medium

Reagent	Final concentration	Amount
StemPro-34 medium	**N/A**	250 mL
StemPro-34 supplement	**N/A**	6.5 mL
L-Glutamine 200 mM	**2 mM**	2.5 mL
Penicillin-Streptomycin	**1%**	2.5 mL
GlutaMax 100×	**1**×	2.5 mL
**Total**	**N/A**	**264 mL**

• To prepare the StemPro34 complete medium, thaw one vial of StemPro-34 supplement at 4°C overnight (approximately 12 h).
  ○ Each bottle of StemPro-34 supplement contains 13 mL.
  ○ Note: Any leftover supplement can be stored at −20°C. In our experience, two freeze-thaw cycles can be tolerated.
• Add the thawed StemPro-34 supplement, L-Glutamine, Penicillin-Streptomycin, and GlutaMax according to the table above.
• StemPro34 complete medium can be stored at 4°C for up to 1 month.

For the culture of SC-ECs, prepare StemPro34 supplemented medium:

***Note:*** To culture one T75 flask of SC-ECs for 1 week, approximately 30 mL of StemPro34 supplemented medium is needed.

**Table:** StemPro34 supplemented medium

Reagent	Final concentration	Amount
StemPro-34 complete medium	**N/A**	42.5 mL
FBS	**15%**	7.5 mL
VEGFA 100 μg/mL	**100 ng/mL**	0.05 mL
FGF2 100 μg/mL	**100 ng/mL**	0.05 mL
**Total**	**N/A**	**50.1 mL**

• To prepare StemPro-34 complete medium with 15% FBS, add 7.5 mL FBS to 42.5 mL StemPro-34 complete medium.

***Note:*** StemPro34 complete medium supplemented with FBS can be stored at 4°C for up to 1 month.

• On the day of use, add VEGFA and FGF2 to the FBS-supplemented StemPro-34 complete medium according to the table above.

***Note:*** When growth factors are added, the medium is stable for 1 day at 4°C.

***Note:*** Use the 100 μg/mL stock solutions for FGF2 and VEGFA prepared in the steps above.

***Note:*** It is recommended that growth factors be added fresh for every medium change and that the stock solutions of VEGFA and FGF2 be stored at −20°C.

### Preparation of the neutralization solution

To prepare a collagen type I hydrogel with a neutral pH (7.4), a neutralization solution is required. This solution should be adapted to the desired final end concentration, stock concentration and specific batch of the soluble collagen. For example, the neutralization solution for a collagen stock concentration of 4.63 mg/mL (Corning, Cat# CLS354236) and a final concentration of 2.5 mg/mL is calculated as follows.

• Prepare a 1 N NaOH solution by dissolving 4.0 g NaOH in 100 mL ddH_2_O.
• Calculate volume (vol) of collagen required for 1 mL.

$$Vol\;of\;collagen\;solution=\frac{Desired\;final\;concentration\times Final\;vol}{Initial\;collagen\;concetration}$$

$$Vol\;of\;collagen\;solution=\frac{2.5\;mg/ml\times 1\;ml}{4.63\;mg/ml}=0.54\;ml$$

• Calculate volume of 10× PBS required for 1 mL.

Volof10xPBS=0.1×Finalvol=0.1×1ml=0.1ml

• Titrate collagen and 10× PBS solution to pH 7.4 using pH-indicator strips.
  ○ Pipette 5 μL onto a strip and compare the color to the reference.
  ○ For our batch, 8 μl of 1N NaOH solution was required.
• Calculate the volume of ddH_2_O required for 1 mL.

Volofwater=Finalvol−(Volofcollagensolution+Volof10xPBS+VolofNaOH)

Volofwater=1ml−(0.54ml+0.1ml+0.008ml)=0.352ml

• Scale up the proportions of 10× PBS, ddH_2_O, and 1N NaOH to obtain the neutralization solution.

**Table:** Neutralization solution

Reagent	Amount
10× PBS	2.174 mL
ddH_2_O	7.652 mL (may vary)
1 N NaOH	0.174 mL (may vary)
**Total**	**10 mL**

• The recipe above was tailored to our specific batch of 4.63 mg/mL collagen.
• Sterile filter the neutralization solution with a 0.2 μm filter.
• Neutralization solution can be stored at 4°C.
• The same neutralization solution can be used on the same batch of collagen for up to a year.

***Note:*** For one chip, 250 μL of collagen solution is required:

$$Vol\;of\;collagen\;solution=\frac{2.5\frac{mg}{ml}\times 0.25\;ml}{4.63\;mg/ml}=0.135\;ml$$

Volofneutralizationsolution=0.250−0.135=0.115ml

### Preparation of EGM-2 complete medium for SC-EC culture on chip

Endothelial growth medium EGM-2 complete medium is used for culturing SC-EC cells on-chip.

**Table:** EGM-2 complete medium

Reagent	Final concentration	Amount
EGM-2 basal medium	**N/A**	495 mL
EGM-2 SupplementMix	**N/A**	12.6 mL
Penicillin-Streptomycin	**1%**	5 mL
**Total**	**N/A**	**506.5 mL**

• EGM-2 complete medium is stable for at least 4 weeks at 4°C.
• EGM-2 SupplementMix can be aliquoted into smaller portions and stored at −20°C for longer periods.

### Preparation of accutase-collagenase P (Accutase-ColP) solution

An Accutase-Collagenase P (Accutase-ColP) solution is used to dissociate cells in the hydrogel chamber and dissolve the collagen type I hydrogels.

• Collagenase P (ColP) stock solution: Prepare a working concentration of 18 U/ml. For example, dissolve 100 mg of Collagenase P with an enzymatic activity of 1.7 U/mg in 9.43 mL of PBS−/−.
  ○ Sterilize by filtering with a 0.2 μm filter.
  ○ Prepare 500 μL aliquots.
  ○ Store stock solutions at −20°C.
  ○ Stock solutions can be stored for up to 6 months.

$$Vol\;of\;PBS=\frac{Weight\;of\;ColP\times enzymatic\;activity}{Desired\;working\;concentration}=\frac{100\;mg\times {1.7\;U.mg}^{-1}}{{18\;U.ml}^{-1}}$$

• To prepare the Accutase-ColP solution, mix one volume of Accutase with an equivalent volume of ColP stock solution (1:1 ratio).
  ○ For 1 mL of Accutase-ColP solution, mix 500 μL Accutase with 500 μL ColP stock solution.
  ○ Keep the solution on ice while working.
  ○ The Accutase-ColP solution should be prepared fresh before every experiment.

### Preparation of 3% agarose solution

• For a 3% agarose solution, mix 0.3 g of agarose with 10 mL of PBS −/−.
• Resuspend the solution and transfer 1 mL into each 2 mL Eppendorf tube.
• Place the tubes in a ThermoMixer (Eppendorf) at 95°C and heat until the agarose is completely dissolved and homogeneous.
• The agarose will solidify when cooled to RT.
• Solidified agarose can be stored in Eppendorf tubes at RT for 2 years.
• For use in the cell extraction process, the agarose can be reheated.

### Preparation of fluorescein isothiocyanate–dextran solution (FITC-dextran solution)

• Dissolve 10 mg of 40 kDa fluorescein isothiocyanate–dextran (FITC-dextran) in 10 mL of PBS −/−
• Sterilize with a 0.2 μm filter.
• Protect from light and store for up to 3 years at 2°C–8°C.

### Preparation of polydimethylsiloxane (PDMS) solution

• Prepare a Polydimethylsiloxane (PDMS) solution by mixing the curing agent and base elastomer at a 1:10 ratio.
• 0.5 g of PDMS is required per chip.
• Prepare a fresh mixture for every chip assembly and use within 3 h or until it becomes too viscous to handle.

## Step-by-step method details

### SC-EC maintenance post-differentiation

**Timing: 1 h**

The following steps describe the maintenance of adherent SC-ECs after successful differentiation. The differentiation of hiPSCs to SC-ECs follows the protocol published by Marder and Remmert et al., 2024.^1^ After differentiation, SC-ECs are cultured on fibronectin-coated tissue culture flasks in StemPro34 supplemented medium. Cells are passaged at 80–90% confluency.

***Note:*** For the seeding of 1 microfluidic chip (seeding of 6 microchannels), one T75 flask with 80–90% confluency should be prepared to obtain a sufficient amount of cells.

***Note:*** This section starts with differentiated SC-ECs. The differentiation of SC-ECs takes 6 days. At least 1 week should be planned for the expansion of SC-ECs after differentiation. Differentiated SC-ECs can be cultured as described below:

1. Prior to passaging, coat tissue culture flasks with a fibronectin coating solution.
2. Use 2 mL of the fibronectin coating solution for a T75 tissue culture flask.
3. Incubate for 30 min at RT.

***Note:*** The fibronectin coating should be done right before every passaging.

4. Aspirate and discard the medium using a serological pipette.
5. Rinse SC-ECs by adding 5 mL of PBS −/− to each T75 tissue culture flask.
6. Discard the PBS.
7. Detach SC-ECs from the plastic by adding 3 mL of Accutase to each T75 tissue culture flask.
8. Incubate for 5 min at 37°C, or until cells visibly detach.
9. Dilute Accutase by adding 2× the volume of medium, then collect the cells into a 15 mL Falcon tube.
10. Centrifuge at 200 × *g* for 5 min at RT.
11. Discard the supernatant and resuspend the cell pellet in 2–3 mL of StemPro34 supplemented medium.
12. Aspirate the fibronectin coating solution from the prepared T75 flask.
13. Reseed cells at a density of 5 × 10^5^ to 7 × 10^5^ cells per T75 flask in 8 mL medium.
14. Change the medium every other day.

***Note:*** Cells can be cultured until passage 6.

***Note:*** SC-ECs can be cryopreserved following standard protocols. To do this, 1 million cells per ml can be cryopreserved in FBS with 10% DMSO.

### Preparation for printing

**Timing: 1 day**

This section highlights all the necessary steps to configure the 3D-printer and make it ready to produce the chips.

***Note:*** For the printing of the chip body, a stereolithography printer based on digital light projection (DLP) is used. However, any printer type and biocompatible resin can be used if the key features are resolvable. A printing resolution of at least 27 μm is recommended.

**CRITICAL:** Before the printing of the 3D chip body, the 3D printer should be configured. Printing parameters are variable between different 3D printers.

15. Configurate the 3D printer.
  a. Our optimized parameters for our chosen resin and 3D printer are:
    i. Burn-in exposure time: 20 s.
    ii. Light intensity: 3.93 mW/cm^2^.
    iii. Layer thickness: 50 μm.
    iv. Anti-aliasing: enabled.

**Figure 5:**
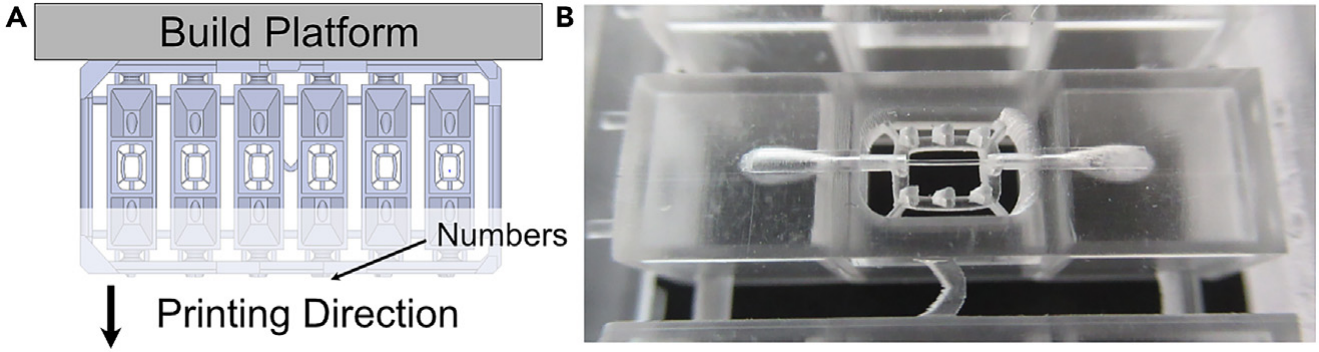
Preparation steps for 3D printing of the chip platform (A) Orientation of chip in the 3D printing process to achieve the highest design resolution. (B) Check if all six pillars are printed and a filament can be fed through the channel. The resolution of these fine features is an indication of a working 3D printer.

**CRITICAL:** Check and adopt printing parameters to resolve all chip features. If using a different 3D-printer, play around with the parameters and print one chip at a time until all channels are open, and the pillar structures are resolved (Figure 5B).

16. Fill the vat to a height of 1 cm with a biocompatible resin, such as GR-10 transparent.
  a. Use a 190 μm filter screen to remove any contaminants from the resin.
17. Download the ‘LumenChip_PrintFile.STL’ file from GitHub.
  a. GitHub: https://github.com/MeierLabMiBioEng/Protocol-to-generate-stem-cell-derived-vessels-on-chip.
  b. Zenodo: https://zenodo.org/doi/10.5281/zenodo.12742938.
18. Load the file into the build software of the 3D printer.
19. Orient the chip so that the side with the numbers is printed as the last layer (Figure 5A).
20. Load as many chips as possible onto the build platform.

***Note:*** The chip is designed to be printed in a vertical orientation, ensuring the microchannel is printed at the highest resolution (Figure 5A). This orientation reduces the risk of clogging the channels and trapping cells on rough surfaces.

**CRITICAL:** Some features will not be printed if printed in a different orientation. Check to see if the pillars are printed and the channel is unblocked as these are the finest features of the chip (Figure 5B).

### Chip production and post-processing

**Timing: 48 h**

In this section, the production and assembly of the microfluidic chip is described.

**Figure 6:**
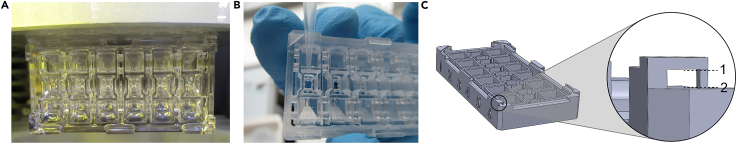
Post-processing of printed chips (A) 3D printed chip with excess resin still attached to the build platform. (B) Use a pipette or syringe to flush out excess resin from the channels with isopropanol if channels are blocked. (C) Clean-up of the support structures after printing. Cut along the lines (1 and 2) before UV flashing to remove the support structure from the chip, allowing the coverslip lid to be inserted.

21. 3D-print the chips.
22. Detach the finished chips from the build platform.
23. Remove as much residual resin as possible using paper towels before washing with isopropanol (Figure 6A).
24. Rinse the excess resin with isopropanol.
  a. This isopropanol bath can be reused for future prints.
  b. If channels are blocked, flush them with isopropanol using a pipette or syringe (see troubleshooting, Figure 6B).
25. Sonicate the chips for 15 min in a new cleaner isopropanol bath.
  a. Ensure all resin is removed.

**CRITICAL:** The chips should not have any wet or glossy spots after drying. Re-sonicate if necessary (Figures 6A and 6B).

26. Incubate the chips at 80°C for 10 min to evaporate any remaining isopropanol.
27. Using a sharp blade or scalpel, carefully cut off the support for the lid.
  a. Be delicate with this fragile structure.
  b. First, cut the top part and then the bottom part as depicted in Figure 6C (see also Figure 1D, reference number 4).
28. Polymerize the chip from both sides using a flash-light polymerization device.
  a. With the Otoflash G171, flash 2000 times on both sides under a nitrogen atmosphere.
29. For biocompatibility, place the chips in 100% ethanol for 24 h.
  a. Make sure to shake off any air bubbles formed.
30. Transfer the chips to a Milli-Q water bath for 24 h.
  a. Make sure to shake off any air bubbles formed.
31. Dry the chips with paper towels.

**Pause point:** Chips can be stored after printing and post-processing at RT in a container, bag, or petri dish.

### Chip assembly

**Timing: 1 h**

This section describes the steps to assemble a 3D-printed chip.

***Note:*** Prepare filaments and adhesive foil as described in the materials and equipment before continuing with the protocol.

**Figure 7:**
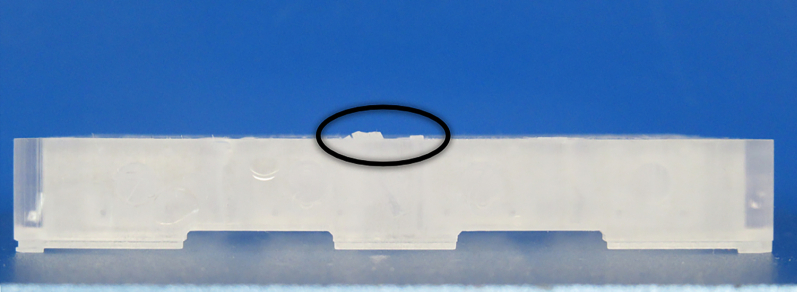
Remove any uneven features on the bottom side using a blade This will prevent the coverslip from cracking when being pressed onto the chip.

**Figure 8:**
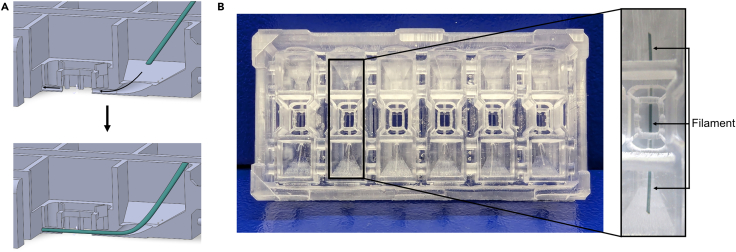
Mounting the chip with the nylon filament (A) Nylon filaments are introduced from one of the two media reservoirs. Be consistent with the direction of insertion. (B) The assembled microfluidic chip with inserted nylon filaments. The filament runs through the hydrogel chamber to create a central lumen after hydrogel polymerization. Within the hydrogel chamber, the 3D-printed insert with support structures stabilizes the hydrogel and serves as a guiding structure for hydrogel removal after the experiment. Support structures at the corners of the 3D-printed body hold the lid in place. For better visualization, a thicker green filament was used for the close-up image.

32. Ensure to cut off any protruding features from the bottom side so that it is flat and smooth (Figure 7, see troubleshooting).
33. Using tweezers, insert the nylon filament from one medium reservoir, through the gel chamber, and out the other medium reservoir (Figure 1D, reference number 3; Figure 8).

**CRITICAL:** Always insert the filaments from the same side and pull them out from the other side after gel filling. This ensures the ‘clean’ tip is pulled through the gel.

***Note:*** Nylon filaments should be inserted before the chip is bonded to the glass coverslip.

**Figure 9:**
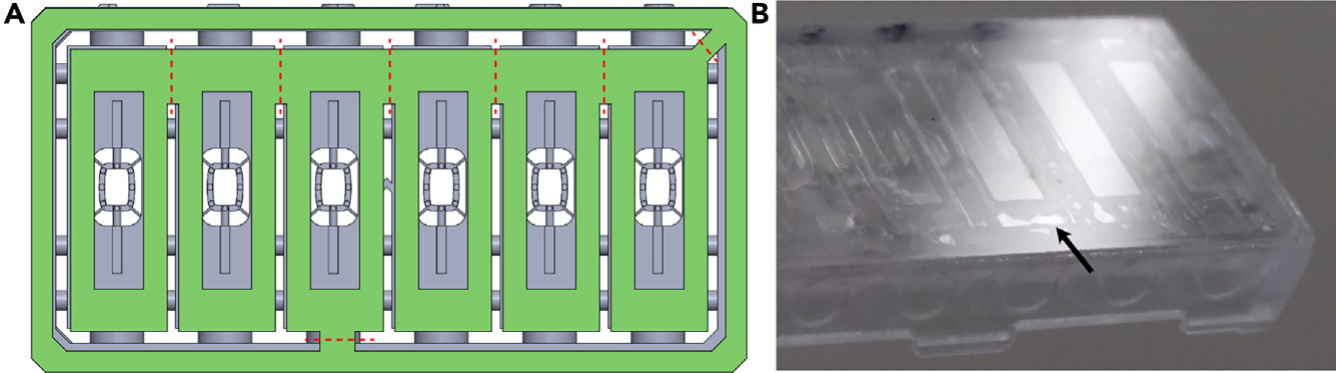
Bonding of the chip to a glass substrate with an adhesive foil (A) Place the adhesive foil (green) onto the bottom of the chip (gray) as depicted and cut along the red lines before pressing the coverslip. Alternatively, cut the foil first and stick the foils onto the chip individually. (B) Make sure the gel chamber is completely sealed off with adhesive foil as depicted. Adhered parts will appear darker. Arrow points to non-adhered part, but the gel is still completely sealed off.

34. Using tweezers, stick the adhesive foil to the bottom side of the chip (Figure 9A).
  a. Cut the areas marked in red to allow individual sealing of every chamber (Figure 9A).

***Note:*** If it makes it easier, you can make the cuts before sticking the foil to the chip.

35. Remove the protective foil to expose the adhesive.
36. Gently but firmly press the glass coverslip onto the foil.
  a. The adhered parts will appear darker (Figure 9B).
  b. Make sure each gel chamber is completely sealed off to prevent PDMS from leaking in.

***Note:*** The chip is about 0.5 mm wider than the coverslip on all sides.

37. Prepare a PDMS solution by mixing the curing agent and base elastomer at a 1:10 ratio (see materials and equipment).

***Note:*** About 500 μL of PDMS solution is required per chip. Higher amounts result in longer curing times but will not affect chip performance.

**Figure 10:**
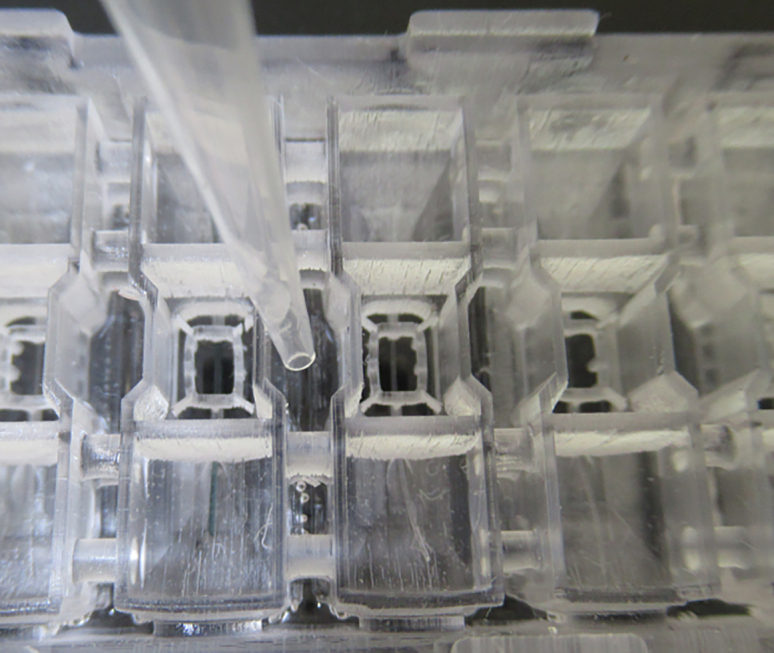
Dispense the PDMS in the gaps between the gel chamber as indicated

38. Dispense the PDMS solution into the gaps between the gel chambers (Figure 1D, reference number 5; Figure 10).
39. Incubate for 30 min at 80°C or overnight at RT.

**Pause point:** Assembled chips can be stored in a dust-free environment at RT for later use.

### Chip sterilization for cell culture

**Timing: 30 min**

**Figure 11:**
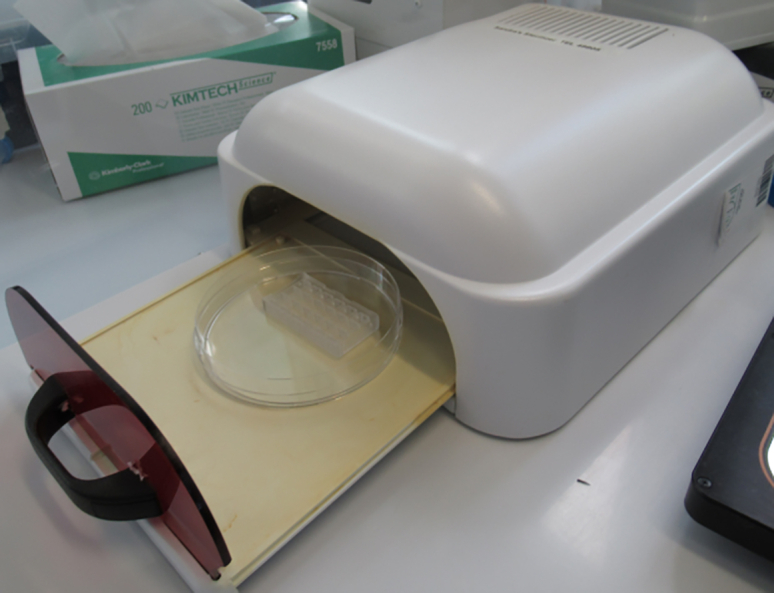
Chip placed in a 10 cm Petri dish in a custom-made UV sterilizer

In this section, the sterilization of chips is performed with a custom-made tabletop UV sterilizer (Figure 11) that can be placed under a cell culture flow hood. However, other UV sterilization devices can also be appropriate.

40. Place assembled chips into a petri dish and open the lid.

***Note:*** A closed lid may affect UV.

41. Sterilize with UV light for 30 min at RT in a sterile work environment.
42. Glass coverslips that function as a lid can also be sterilized with UV light.
43. Store chips in a sterile environment at RT.

### Hydrogel filling into microfluidic chip

**Timing: 2 h**

This section describes the production of a microchannel in a collagen type I hydrogel for seeding SC-ECs on the chip. For this step, use sterile, assembled chips with inserted nylon filaments.

***Note:*** This protocol details the production of chips with a 2.5 mg/mL collagen type I hydrogel. However, other collagen concentrations or different hydrogels can also be used (see Marder and Remmert et al.^1^).

44. Prepare the neutralization solution according to the instructions in the materials and equipment section.
45. Cool the chips for hydrogel filling at −20°C for 1 h in a petri dish.
46. In the meantime, prepare a collagen type I hydrogel with a final concentration of 2.5 mg/mL using the neutralization solution.
  a. For 6 gel chambers, prepare 250 μL of collagen gel.
  b. By using a 100 μL pipette, mix thoroughly by stirring and pipetting up and down.

***Note:*** The volumes of the collagen stock solution and the neutralization solution may vary between different collagen batches. When using a new collagen batch, adjust the pH to 7.4 and modify the neutralization solution accordingly as described in the materials and equipment section.

**CRITICAL:** Work on ice.

***Note:*** Prevent bubbles when mixing the hydrogel. In case of bubbles, centrifuge the hydrogel solution in a precooled centrifuge (4°C) at 300 g for 1 min (see also troubleshooting).

**Figure 12:**
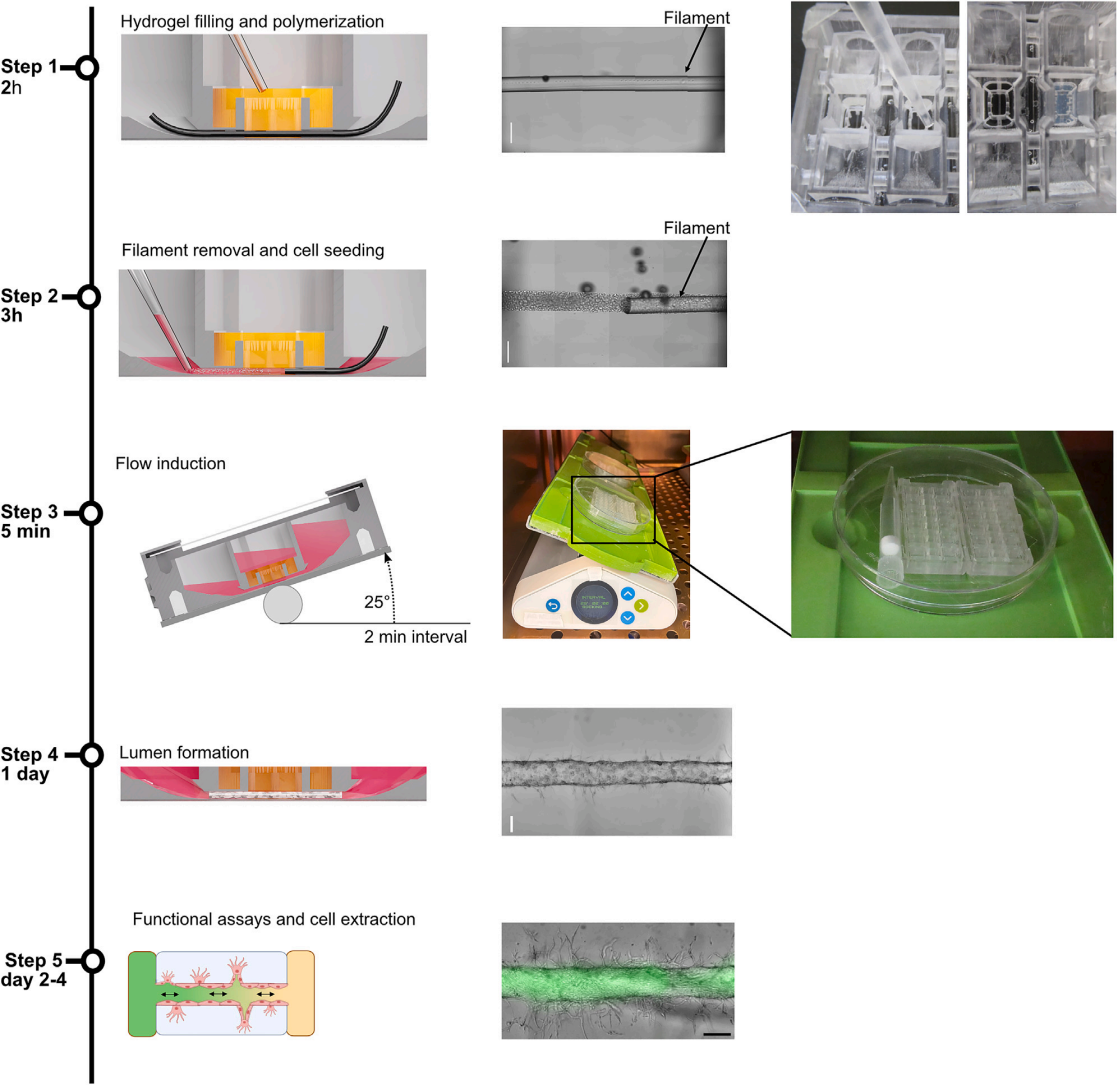
Schematic and bright-field images of cell seeding procedure on chip Fill the gel chamber with hydrogel and induce cross-linking (step 1). After placing the cell suspension in the inlet and outlet, remove the filament to introduce cells into the channel (step 2). Place two chips in a 10 cm Petri dish and secure them in place with a 1000 μL pipette tip or any other object of the appropriate size. Following incubation, wash out non-adherent cells and place the chip on a rocking shaker to induce flow (step 3). Vascular lumens are formed after 1 day of culture (step 4). Functional assays, such as FITC-dextran perfusion to test for barrier function, and cell extraction for downstream analysis can be performed after 2 days of culture on the chip. The culture time can be extended according to the research question (step 5). Scale bar: 200 μm.

47. Incubate the prepared collagen solution on ice at 4°C for 1 h.
48. After incubation, fill the gel chamber with the collagen gel (40 μL per chamber) (Figure 1D, reference number 2; Figure 12, step 1).
  a. After filling, tap the chips on a flat surface to ensure the gel fills the entire gel chamber.

**CRITICAL:** Place the chips on ice during pipetting.

**CRITICAL:** Prevent bubbles in the hydrogel by pipetting slowly and only until the first pause point of the pipette.

49. For collagen polymerization, incubate the chips in a cell culture incubator at 37°C with 5% CO_2_ for 40 min.

**CRITICAL:** Transfer the chip directly into the cell culture incubator after filling it with hydrogel. Do not place the chips at RT, as this can affect hydrogel polymerization.

### Seeding of SC-ECs into the microfluidic chip

**Timing: 3 h**

This step describes the seeding of SC-ECs into the pre-patterned microchannel. For the cultivation on the chip, the endothelial growth medium EGM-2 complete is used. The preparation of the cell suspension can be done while the collagen type I hydrogel polymerizes in the chip’s gel chamber.

***Note:*** The day of seeding is referred to as day 0.

50. Place a rocking shaker (OrganoFlow L) in a cell incubator (37°C, 5% CO2) under sterile conditions for later chip culture.
51. Detach SC-ECs as described above (steps 4–10).
52. Resuspend the cell pellet in 1–5 mL of EGM-2 complete medium.
53. Count cells using the TC20 automated cell counter or a hemocytometer.
54. Adjust the cell concentration to 4 million cells per mL in an Eppendorf tube.

***Note:*** Cells can be kept at RT.

55. Resuspend the cells thoroughly.
56. Place chips with polymerized hydrogel under a sterile flow hood.
57. Add 20 μL of the resuspended cell suspension into the inlet and outlet chambers (Figure 1D, reference number 1).

***Note:*** If the cell suspension is limited, the volume can be reduced to 10 μL per inlet and outlet chamber.

**CRITICAL:** Resuspend the cell suspension immediately before seeding to avoid cell clumps, which can block the channel (see also troubleshooting).

58. Pull out the filament with sterile forceps to create a gravity-driven flow that pulls the cell suspension into the patterned microchannel (Figure 12, step 2, and Methods video S1).

**CRITICAL:** To remove the filament properly, pull it in one smooth motion.

***Note:*** We recommend seeding no more than 3 microchannels at a time and then checking the success of the seeding. Like this, the seeding process can be adjusted if necessary. If the seeding outcome is insufficient (see Figure 15 and troubleshooting), the volume and concentration of cell suspension might need adaptation.

59. Check for successful seeding under a microscope (see troubleshooting and Figure 15).
60. After seeding, place the chip in a sterile petri dish.
61. Place the chips on the rocking shaker at a 10° angle and 6 rpm speed for 15 min to allow cell attachment to the hydrogel walls (Figure 12, step 3).
  a. Two chips can be placed inside a 10 cm petri dish and secured by placing an additional item, such as a 1000 μL pipette tip.
62. Incubate for 15 min.
63. Add 30 μL of EGM-2 complete medium into the inlet, outlet, and hydrogel chambers (Figure 1D, reference numbers 1 and 2).
64. Close the chip with a glass coverslip.
65. Incubate for 2 h on the shaker at a 25° angle with 2-min intervals (Figure 12, step 3).

***Note:*** Shear flow can be adjusted by changing the angle of the rock shaker (see Marder and Remmert et al.^1^).

**CRITICAL:** Adjust the position of the chips on the shaker to ensure the medium can flow through the generated lumen (Figure 12, step 3).

66. Observe the cell attachment of SC-ECs to the hydrogel walls under a microscope.
67. Incubate for 2 h.
68. After 2 h of incubation, completely change the medium to remove excess cells.
  a. Aspirate the medium from every chamber with an aspiration pipette.
69. Add 70 μL of medium to each chamber.

***Note:*** For this step, a multi-pipette such as the Multipette (Eppendorf) can be used for faster medium changes.

70. Close the chip with a glass coverslip.
71. Place the chips back on the rocking shaker.
  a. Setting as in step 65.
72. Change the medium daily.
73. Observe the formation of an endothelial vessel-on-chip.

***Note:*** After 2–4 h after seeding, SC-ECs should have attached to the microchannel walls and changed from a round to a flat morphology. By day 1, the first signs of sprouting angiogenesis should be observed (Figure 12, steps 4 and 5).

Methods video S1. Demonstration of nylon filament removal after hydrogel filling and polymerization, related to step 58

### Lumen permeability analysis

**Timing: 1 h**

This section describes the assay for testing the permeability of the formed vessels-on-chip.

74. In a sterile environment, remove the media from the two media reservoirs.
75. Add 50 μL of 40 kDa FITC-dextran solution to one reservoir.
76. Place the chip on a fluorescence microscope.
77. Set up a time series with 3 min intervals for a total of 20 min.
  a. Select a bright field as well as a FITC channel (488 nm).
78. Start the time series.

***Note:*** The images will reveal any leakages if a barrier function is not present.

79. In a sterile environment, remove the FITC-dextran.
80. Flush the channels once with 100 μL PBS −/− to remove residues.
81. Place media into the media reservoirs (70 μL per reservoir).
82. Return the chips to the shaker and incubator (see step 65).

***Note:*** It is recommended to use a stage top incubator, such as the STX from Tokai Hit, during longer imaging procedures.

### Extraction of single cells from the vessel-on-chip for further analysis

**Timing: 2 h**

This section describes the extraction of single cells from the vessel-on-chip for further analysis. This enables the examination of vascular structures using state-of-the-art technologies such as scRNA-seq or proteomics. Since the seeding process causes cells to grow in the plastic channels of the inlets and outlets, a special extraction technique must be used to isolate only cells from the hydrogel. To achieve this, agarose can be filled into the inlet and outlet chambers to block the connection between the hydrogel chamber and the plastic part. Like this, only the cells from the hydrogel chamber can be extracted.

83. Preheat a 3% agarose solution at 95°C and 1000 rpm in a ThermoMixer (Eppendorf).
84. Rinse the cells on the chip by adding 70 μL PBS −/− to each inlet, outlet, and hydrogel chamber.
85. Aspirate the PBS from the chip chambers and discard it.
86. Block the inlet and outlet ports with 20 μL of pre-warmed agarose (95°C).

***Note:*** As the agarose solution solidifies within seconds, it needs to be pipetted directly from the ThermoMixer (Eppendorf) into the ports.

87. Prepare an Accutase-ColP solution on ice (materials and equipment).
  a. To dissolve hydrogels in 6 chambers, mix 210 μL of ColP with 210 μL of Accutase.

***Note:*** The Accutase-ColP solution is need for the digestion of the collagen type I hydrogel and the generation of a single-cell suspension.

88. Add 70 μL of the Accutase-ColP solution on top of each hydrogel.
89. Incubate for 30 min at 37°C with 5% CO_2_ or until the hydrogel is visibly dissolved.

**CRITICAL:** It is important that the hydrogel is completely dissolved and the vascular structures dissociate into single cells. If a single-cell suspension is not achieved after 30 min, prolong the incubation time. Careful pipetting can also help dissociate the cells but avoid high shear stress as it can damage the cells. Prefer prolonged incubation times over mechanical dissociation.

90. Collect the cell suspensions in Eppendorf tubes.
91. Stop the reaction by adding an equal volume of PBS + 2% BSA.

***Note:*** To ensure the collection of all cells, the gel chambers can be washed with PBS + 2% BSA. Check under a microscope for a successful collection of cells. The maximum yield of cells per lumen is approximately 3000 cells. Make sure to collect as much cells as possible to provide sufficient cellular input for downstream analysis. Depending on the downstream analysis, the necessary cell concentration will vary.

92. Centrifuge at 300 × *g* for 3 min, at RT.
93. Discard the supernatant.
94. Resuspend in 500–1000 μL PBS + 2% BSA.
95. Repeat steps 92–94.
96. Count the cells (see step 53).
97. Use the cell suspensions for downstream analysis such as scRNA-seq, proteomic analysis, or other functional assays (see Marder and Remmert et al.^1^).

**CRITICAL:** For scRNA-seq protocols, a cell viability of >80% is strongly recommended.

### Extraction of vascular tissue for sectioning and histological analysis

**Timing: 1 h**

This section describes the retrieval of vascular structures for sectioning and histological analysis. To analyze the endothelial structures, the vessels including the hydrogel can be fixed and removed from the chip. Retrieved hydrogels are embedded for sectioning and histological analysis.

**Figure 13:**
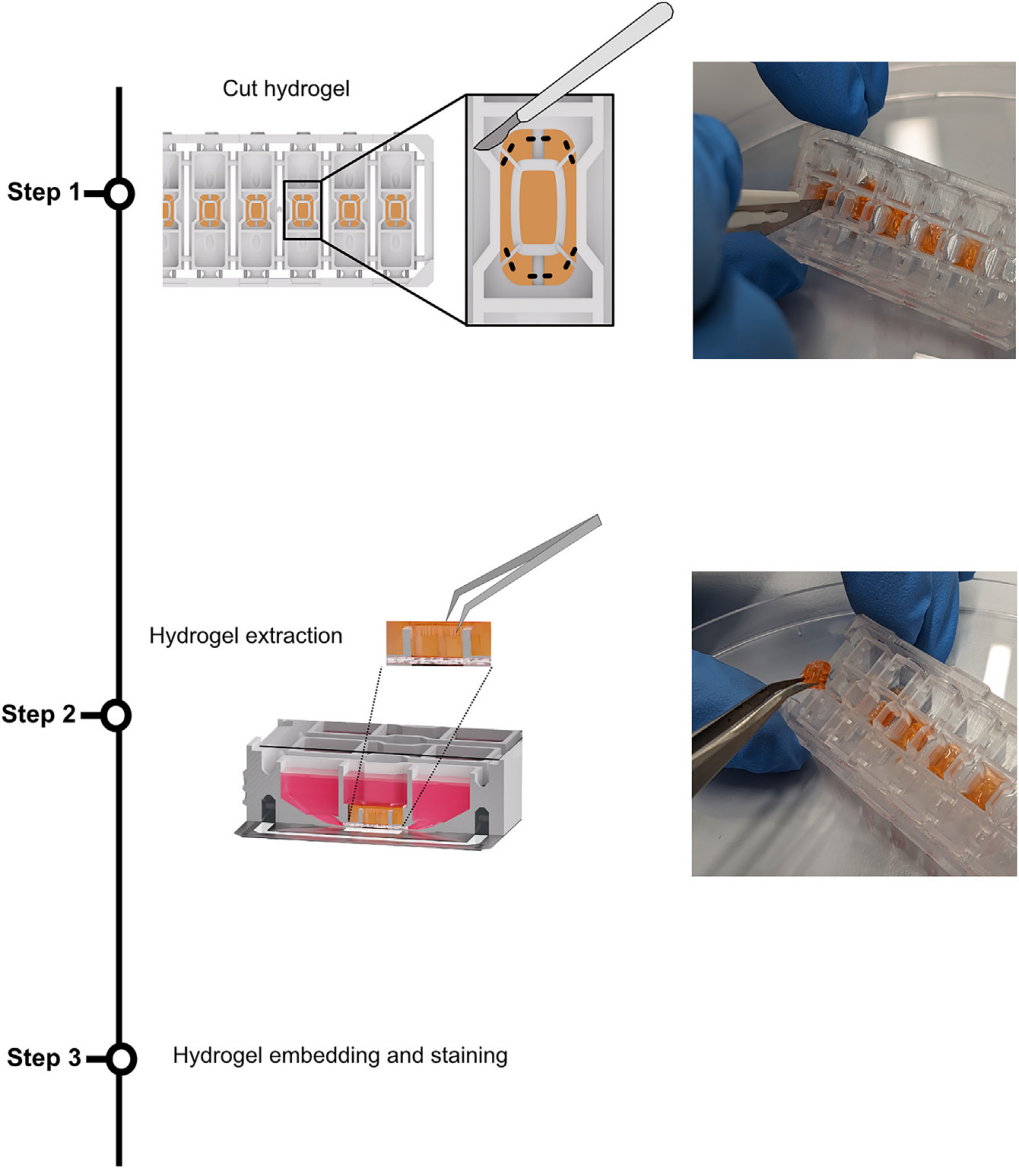
Retrieval of the hydrogel with the tissue from the chip for histological analysis To extract the vascular structures within the hydrogel, the support structures of the hydrogel chamber need to be cut with a scalpel (step 1). The best results are obtained using a no. 11 scalpel with an elongated triangular blade. After cutting, the hydrogel block can be extracted with tweezers (step 2) and further processed for embedding and staining (step 3).

98. Rinse the cells on the chip by adding 70 μL PBS −/− to each inlet, outlet, and hydrogel chamber.
99. Aspirate the PBS and discard it.
100. Fix the cells by adding 70 μL of Roti Histofix (4% PFA) to each inlet, outlet, and hydrogel chamber.
  a. Incubate for 30 min at RT.
  b. Wash the cells by repeating steps 98 and 99.
101. Cut the rated breakpoints of the scaffolding structure with a scalpel (Figure 13, step 1).
  a. For best results, use a no. 11 scalpel, which has elongated triangular blades with narrow tips, making cutting easier.

**CRITICAL:** When cutting, care should be taken to cut only at the edge of the hydrogel. It is recommended to use a microscope while cutting. Check for successful cutting under a microscope.

102. Once all plastic parts are cut, lift the scaffolding structure with tweezers (Figure 13, step 2).

**CRITICAL:** To ensure that all plastic parts are cut sufficiently, the hydrogel can be lifted slightly with tweezers. If it does not come out of the chamber easily, cut again.

103. Transfer the extracted gel chambers into Eppendorf tubes containing PBS −/−.
104. Store them at 4°C for further processing.
105. Perform the standard embedding procedure for immunohistochemistry staining according to your laboratory’s protocol (Figure 13, step 3, see Marder and Remmert et al.^1^).

***Note:*** Samples can be used for paraffin embedding or cryosectioning.

## Expected outcomes

**Figure 14:**
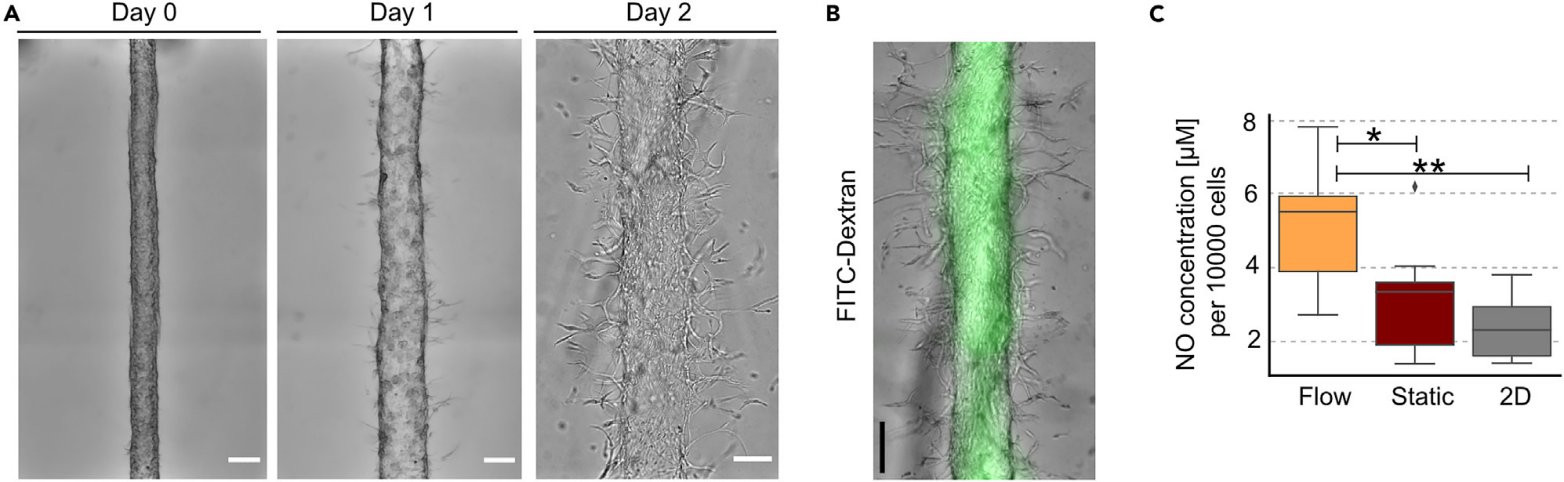
Representative vessels formed on the microfluidic chip (A) Bright-field images of the vascular lumen immediately after seeding and after 1 or 2 days of culture under shear flow conditions. Sprouting angiogenesis of SC-ECs is evident from day 1. Scale bar: 200 μm. (B) Endothelial barrier formation was analyzed by perfusing the vessels with FITC-Dextran (green). Scale bar: 200 μm. (C) Nitric oxide (NO) concentrations were measured from aliquots collected from the reservoirs. Supernatants of SC-ECs cultured on-chip under shear flow conditions showed higher NO concentrations compared to those cultured under static conditions on-chip or in 2D monolayer cultures (n = 8–9 from 3 independent experiments; error bars indicate 95% CI). NO concentrations were normalized to the total cell count per sample. Therefore, single cells were extracted from the lumen as described above. *p*-values were calculated using a parametric one-way ANOVA and Tukey’s *post hoc* test (∗*p* ≤ 0.05, ∗∗*p* ≤ 0.01). Figure reprinted and adapted with permission from Marder and Remmert et al., 2024.^1^

This protocol enables the engineering of stem cell-derived vessels-on-chip. Vascular lumens form within one day and can be placed under gravity-driven flow for extended cell culturing periods. After two days under shear flow conditions, SC-ECs exhibit sprouting angiogenesis. The engineered vessels form an endothelial barrier, which can be analyzed by perfusing the vessels with fluorescent-labeled FITC-Dextran (Figures 14A and 14B). In addition, SC-ECs under shear flow show increased nitric oxide (NO) synthesis, a hallmark of functional endothelial cells, compared to 2D and static control groups (Figure 14C).^5^

The developed vessel-on-chip platform can be analyzed with state-of-the-art cell technologies, such as single-cell RNA sequencing (scRNA-seq), proteomics, and secretome analysis by mass spectrometry, as well as time-lapsed high-resolution imaging. In Marder and Remmert et al.,^1^ we reported that SC-ECs mature under flow, demonstrating barrier formation, arterial toning, and high NO synthesis. By using either hiPSCs with genomic predispositions or stimulating healthy SC-ECs with disease-related factors, a vascular disease model can be established. We demonstrated that SC-ECs responded to stress factors known to induce atherosclerosis, leading to altered NO synthesis and expression of oxidative stress genes. Combined with the open hydrogel design and the manufacturing process via 3D printing, our setup facilitates the engineering of more complex and custom tissues.

## Limitations

This vessel-on-chip system generates bidirectional flow without the use of a pump. Under physiological conditions, blood flow is unidirectional, with wall shear stresses varying significantly depending on organ function, vessel size, and the viscoelastic properties of blood. For instance, shear rates in capillaries or lymphatic vessels are around 2 dyne/cm^2^, while in larger arteries, they range from 10 to as high as 70 dyne/cm^2^.^6^^,^^7^ In our chip system, gravity-driven flow results in wall shear stress within the lower physiological range, at 3 dyne/cm^2^.^1^ Additionally, endothelial cells (ECs) degrade the collagen hydrogel over time, hindering long-term cultures. To facilitate long-term cultures of 7 days and longer, the hydrogel must be adjusted to be stiffer and more stable.

## Troubleshooting

### Problem 1

Uneven surfaces and small print errors protruding from the chip prevent the adhesive foil from creating a tight seal. As a result, the glass coverslip breaks during chip assembly, causing the PDMS to leak into the gel chambers (related to step 32).

### Potential solution

• Use a blade or scalpel to trim any protrusions that are making the bottom of the chip uneven.
• Apply additional pressure to ensure the adhesive foils are properly adhered. Properly adhered regions will appear darker (see Figure 9B).

### Problem 2

Air bubble formation in the hydrogel after pouring in the hydrogel chamber (related to steps 45, 46, 48, and 32).

### Potential solution

• Cool the chips to −20°C and place them on ice before pipetting the hydrogel to prevent rapid polymerization.
• Avoid air bubbles by gently pipetting and resuspending the hydrogel.
• Pipette the hydrogels into the gel chamber without introducing air bubbles. While pipetting, place the pipette tip inside the hydrogel chamber and fill at a slow speed until the first stop of the pipette.
• If air bubbles appear in the hydrogel after 1 or 2 days of culture, they may originate from air trapped between the adhesive foil, the chip’s 3D-printed part, and the glass coverslip. To avoid trapped air, ensure that the adhesive foils are properly adhered to both the 3D-printed part and the glass coverslip during assembly.

### Problem 3

**Figure 15:**
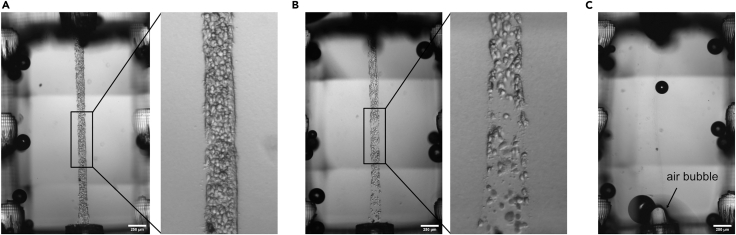
Expected seeding outcome and troubleshooting Bright-field images of vascular lumen right after seeding with (A) optimal seeding density, (B) a too low seeding density, or (C) a failed seeding because of an air bubble. Scale bar, 250 μm.

Microchannels are blocked after hydrogel pouring and hinder the seeding of cell suspension in the microchannel (related to steps 24, 55–59) (Figure 15).

### Potential solution

• Clogged channels can be reduced by flushing the channels with isopropanol during the post-processing steps if this frequently occurs.
• Cell clumps can block the microchannels: To avoid seeding cell clumps, thoroughly resuspend the cell suspension.
• Cell suspension is not entering the microchannel: Incomplete seeding may also indicate that not enough cell suspension is entering the microchannel. Therefore, it is important that the cell suspension is pipetted directly into the inlet and outlet openings.
• Air bubbles stuck in the opening of the channel can block the lumen. Using a 10 or 2.5 μL pipette, aspirate media, insert the tip into the channel opening, and gently push approximately 0.5 μL of suspension through the channel. Increase the force and volume until the channel gets unclogged. Observe the level of the meniscus inside the pipette tip as an indicator to see if anything is going through. The passing of the air bubble through the channel will also be visible. Pushing too hard at once will cause the channel to burst so start gently.
• Use a 10 or 2.5 μL pipette to try to pull out any air bubbles from the inlet and outlets before pulling the filament out.
• Using a different cell line can influence the seeding procedure: If so, adjust the seeding density. A blockage of the microchannels can indicate that the cell concentration is too high.

### Problem 4

Microchannel seeding density is too low (related to steps 24, 55–59) (Figure 15).

### Potential solution

• Cell suspension needs to be concentrated.
• Microchannels might be blocked, see problem 3.

### Problem 5

After single-cell extraction from the microfluidic chip, the cell viability is below 70% (related to step 89).

### Potential solution

• Applied shear stress for the single cell extraction was too high: Prolonged incubation times should be preferred to mechanical dissociation. Using a stronger dissociation reagent instead of Accutase is recommended if no single-cell suspension is achieved after 40–60 min.

## Resource availability

### Lead contact

Further information and requests for resources and reagents should be directed to and will be answered by the lead contact, Matthias Meier (matthias.meier@helmholtz-munich.de).

### Technical contact

Further information and requests regarding the chip design and engineering questions can be directed to Munkhtur Otgonbayar (munkhtur.otgonbayar@helmholtz-munich.de). Requests and information regarding the biological application should be directed to Caroline Remmert (caroline.remmert@helmholtz-munich.de).

### Materials availability

The STL and SVG files of the chip and the adhesive foils, respectively, are available for download from our GitHub account (https://github.com/MeierLabMiBioEng/Protocol-to-generate-stem-cell-derived-vessels-on-chip; https://doi.org/10.5281/zenodo.12742938).

### Data and code availability

All datasets and functional analyses on the vessel-on-chip are published in Marder and Remmert et al.^1^

## Acknowledgments

This work was supported by the Helmholtz Pioneer Campus, the eISLET project of the 10.13039/501100002347BMBF (grant number 031L0251), and an 10.13039/100010663ERC Consolidator Grant (number 772646). We thank Michel Moussus for helping with the initial design of the chip platform. We thank Stefan Veit, Revanth Coimbatore Varadarajan, and Che-Wei Chang for the manufacturing of the chips. We thank Flavien Martinot and Christoph Müthering for helping with the images of the chip.

## Author contributions

C.R. and M. Marder executed biological experiments and established biological readouts on the chip. M.O. and J.A.P. designed and characterized the chip. The manuscript was written by C.R., M.O., and M. Meier. All authors corrected and approved the paper.

## Declaration of interests

J.A.P., M.O., and M. Meier filed a patent application for the organ-on-chip technology described herein.
